# Is sleep bruxism related to the levels of enzymes involved in the serotonin synthesis pathway?

**DOI:** 10.1007/s00784-021-04329-1

**Published:** 2021-12-09

**Authors:** Joanna Smardz, Helena Martynowicz, Anna Wojakowska, Joanna Wezgowiec, Cyprian Olchowy, Dariusz Danel, Grzegorz Mazur, Mieszko Wieckiewicz

**Affiliations:** 1 Department of Experimental Dentistry, Wroclaw Medical University, Wroclaw, Poland; 2 Department and Clinic of Internal Medicine, Occupational Diseases, Hypertension and Clinical Oncology, Wroclaw Medical University, Wroclaw, Poland; 3 Department of Anthropology, Hirszfeld Institute of Immunology and Experimental Therapy, Polish Academy of Sciences, Wroclaw, Poland; 4 Department of Dental Surgery, Wroclaw Medical University, Wroclaw, Poland

**Keywords:** Sleep bruxism, Masticatory muscle activity, Serotonin, Tryptophan hydroxylase, Aromatic l-amino acid decarboxylase, DOPA decarboxylase, Neurotransmission

## Abstract

**Objectives:**

This exploratory research aimed to evaluate the levels of tryptophan hydroxylase 1 (TPH1) and aromatic l-amino acid decarboxylase (DDC), which play an important role in the serotonin synthesis pathway, in individuals with sleep bruxism (SB) diagnosed using audio–video polysomnography (vPSG) and compare them with that of individuals not presenting with SB.

**Materials and methods:**

The study included adult patients hospitalized in the Department and Clinic of Internal Medicine, Occupational Diseases, Hypertension and Clinical Oncology at the Wroclaw Medical University. The participants underwent a single-night vPSG for the evaluation of the SB parameters. Peripheral blood samples were also collected from them for estimating the serum levels of TPH1 and DDC.

**Results:**

A total of 105 patients (80 women and 25 men) were included in the study. All the patients were Caucasians and aged 18–63 years (mean age: 33.43 ± 10.8 years). Seventy-five patients (71.43%) presented with SB, of which 50 (47.62%) had severe SB, while the remaining 30 patients (28.57%) did not. No statistically significant differences in TPH1 and DDC levels were observed between the individuals with SB and without SB. A significant negative correlation was found between tonic SB episodes and DDC levels (*p* = 0.0012). Other correlations between the SB parameters and the levels of the studied enzymes were statistically insignificant (*p* > 0.05 for all comparisons).

**Conclusions:**

The levels of the enzymes that are crucial for serotonin synthesis (TPH1 and DDC) did not seem to influence SB.

**Clinical relevance:**

This study provides important insights for further research on the relationship between the serotonin pathway and SB, which should take into account not only the process of serotonin synthesis but also the effect of serotonin-dependent neurotransmission on SB.

## Background

SB is a common motor behavior currently defined as “the activity of masticatory muscles during sleep, which may be rhythmic (phasic) or nonrhythmic (tonic) and is not a movement disorder or a sleep disorder in otherwise healthy individuals” [1]. It is estimated that approximately 13% of the adult population is affected by SB [1, 2]. Many bruxers are unaware of their condition, and thus, the actual incidence of SB can be possibly underestimated [3]. However, SB may have harmful clinical implications, such as damage to dental hard tissues, prosthetic restorations, and oral mucosa, as well as orofacial pain [1–4]. Furthermore, SB can co-occur with other serious conditions such as sleep-related breathing disorders [5, 6], cardiovascular diseases [7], psychoemotional disturbances [1–4, 8], insomnia [9], and changes in the sleep structure [10]. SB is also considered a major diagnostic challenge for clinicians due to its distinct circadian manifestation [1–4].

SB has been shown to be associated with three types of factors: biological, psychological, and exogenous. One of the biological factors is neurotransmission [1, 2]. Our previous study indicated that the genes responsible for the serotonin neurotransmission pathway may be involved in the SB pathogenesis [11]. Moreover, the participants of the study who presented with severe SB had lower levels of serotonin which was analyzed in a further study. In the context of the relationship between the serotonin pathway and bruxism, it should be noted that SB can be modulated by the intake of serotonergic antidepressants, including selective serotonin reuptake inhibitors (SSRIs) and serotonin-norepinephrine reuptake inhibitors (SNRIs).

Serotonin (5-hydroxytryptamine, 5-HT) is a monoamine neurotransmitter of the central nervous system, which is synthesized from tryptophan obtained from dietary sources [12]. During the synthesis, tryptophan is converted to 5-hydroxytryptophan (5-HTP) [13] by biopterin-dependent monooxygenation catalyzed by tryptophan hydroxylases 1 and 2 (TPH1 and TPH2), and then 5-HTP is decarboxylated by aromatic l-amino acid decarboxylase (DDC) to 5-HT [14]. However, the cause of the decreased serotonin levels in patients with severe SB is not known yet. However, the fact that SB can be worsened by the intake of serotonergic antidepressants, including SSRIs and SNRIs, cannot be excluded [15]. Therefore, there is a need to analyze which stage of the serotonin pathway is responsible for this relationship. Etzel et al. reported that L-tryptophan supplementation did not cause any significant differences in bruxing levels in patients with SB [16]. This suggests that another stage of the pathway may be responsible for the decreased serotonin level in patients with severe SB, and perhaps, the level of enzymes responsible for serotonin synthesis may be the reason. Thus, this exploratory research was conducted to evaluate the levels of TPH1 and DDC in individuals with SB diagnosed using vPSG [1–4] and compare them with that of individuals not presenting with SB.

## Methods

The participant recruitment, inclusion, and exclusion criteria, as well as the vPSG procedure, parameters, and scoring are partially described in our previous papers [5–7, 10, 11, 17].

### Participants

The participants included in this study were adult patients hospitalized in the Department and Clinic of Internal Medicine, Occupational Diseases, Hypertension and Clinical Oncology at the Wroclaw Medical University. The study was conducted following the guidelines of the Declaration of Helsinki and approved by the Ethical Committee of the Wroclaw Medical University (ID: KB-794/2019). All participants provided written informed consent. Information regarding the clinical trial registration can be found at www.ClinicalTrials.gov (identifier: NCT04214561).

### Inclusion criteria

The inclusion criteria of the study were as follows: age ≥ 18 years, clinical diagnosis of SB based on the International Consensus on the Assessment of Bruxism [1], and willingness to participate in the study.

### Exclusion criteria

The exclusion criteria included the following: severe systemic disorders and diseases (including genetic disorders); neurological disorders and/or neuropathic pain; active inflammation; active malignancy; severe mental disorders and significant mental (including genetic) disabilities; pregnancy and confinement; treatment with or addiction to any analgesic agents and/or drugs that can affect the functions of the nervous system, muscles, and respiratory system; lack of agreement to participate in the study.

### Recruitment

The participants were recruited from the Outpatient Clinic of Temporomandibular Disorders operating in the Department of Experimental Dentistry at the Wroclaw Medical University. After recruitment, the patients were subjected to a thorough medical interview and an intra- and extraoral examination based on the Diagnostic Criteria for Temporomandibular Disorders [18] through self-reporting (including reporting by bed partner) and assessed for the signs and symptoms of bruxism by an experienced dentist. Those diagnosed with probable SB based on the Third Edition of the International Classification of Sleep Disorders by the American Academy of Sleep Medicine [19] were subjected to a single-night vPSG analysis.

### Polysomnography

vPSG was carried out using NoxA1 (NOX Medical, Reykjavík, Iceland) in the Sleep Laboratory operating in the Department and Clinic of Internal Medicine, Occupational Diseases, Hypertension, and Clinical Oncology at the Wroclaw Medical University. Taking into account the sleeping habits and individual preferences of the patients, the recordings were obtained from 10:00 pm to 06:00 am. The electrodes were arranged as recommended by the manufacturer, except that the bipolar leads for electromyographic recording were placed from both sides of the origin and insertion of the masseter muscles.

During vPSG, the following standard elements were examined: electroencephalographic, electrocardiographic, electrooculographic, and electromyographic recordings from the chin area and bilaterally from the masseter muscles; recordings of abdominal and thoracic breathing activity; body position; and audio–video recordings. The saturation level, pulse, and plethysmographic data were recorded with a NONIN WristOx2 3150 pulse oximeter (Nonin Medical Inc., Plymouth, MN, USA). The entire vPSG record was restored using Noxturnal software (Nox Medical, Reykjavík, Iceland). All the obtained vPSG recordings were scored and analyzed in 30-s epochs by a qualified and experienced physician in accordance with the 2013 American Academy of Sleep Medicine standard criteria for sleep scoring [20].

#### SB parameters

The diagnosis of SB was performed using bilateral masseter electromyography (EMG) and audio–video recordings. The following indices were assessed during the analysis: bruxism episodes index (BEI), phasic bruxism (increases in more than three cyclic phasic EMG signals lasting 0.25–2 s), tonic bruxism (episodes lasting > 2 s), and mixed bruxism. The new SB episodes were scored after at least 3 s of stable EMG and when the activity was at least twice the amplitude of the background EMG [19, 20]. Furthermore, based on the number of bruxism episodes per hour of sleep (BEI), SB was classified as irrelevant (BEI < 2), mild to moderate (BEI = 2–4), or severe (BEI > 4) [19].

### Enzyme level assessment

For analyzing the levels of enzymes involved in the serotonin synthesis pathway, 4 mL of peripheral blood was collected from an antecubital vein from each patient using the Vacutainer® (Becton Dickinson, Franklin Lakes, NJ, USA) blood sampling system. Then, the blood samples, which were collected into anticoagulant-free tubes, were centrifuged at 4500 × *g* for 10 min to obtain serum The serum samples were transferred to 1.5-mL Eppendorf tubes (Eppendorf, Hamburg, Germany), frozen, and stored at –80 °C until the day of analysis.

#### TPH1 serum level measurement

The serum level of TPH1 was analyzed using a commercially available enzyme-linked immunosorbent assay (ELISA) kit (ref. E-EL-H5313; Elabscience, Houston, TX, USA) which had a sensitivity of 0.19 ng/mL and a detection range of 0.31–20 ng/mL. The procedure was carried out based on the basic principle of Sandwich ELISA, according to the manufacturer’s instructions. After optimization of the laboratory procedure, the serum samples were diluted tenfold with the sample diluent in order to match the measured values to the detection range of the ELISA kit. The optical density (OD) was measured using a microplate reader (Multiskan GO; Thermo Fisher Scientific, Waltham, MA, USA) at 450 nm. Standard curve fitting was performed with GraphPad Prism (GraphPad Software, San Diego, CA, USA), and then the concentrations of samples were quantified. Each sample was measured in triplicate, and the mean serum levels of TPH1 were calculated for each patient.

#### DDC serum level measurement

The serum level of human DDC was analyzed using a commercially available ELISA kit (ref. ELH-DDC-1; RayBiotech, Peachtree Corners, GA, USA) with a sensitivity of 34 pg/mL and a detection range of 34–8000 pg/mL. The assay was based on Sandwich ELISA and performed according to the manufacturer’s instructions. The serum samples were diluted twofold using the assay diluent. OD was measured using a microplate reader (GloMax® Discover; Promega, Madison, WI, USA) at 450 nm. Standard curve fitting was performed using GraphPad Prism (GraphPad Software, San Diego, CA, USA), and then the concentrations of samples were quantified. Each sample was measured in triplicate, and the mean serum levels of DDC were calculated for each patient.

### Data analysis

The obtained data were analyzed using Statistica 13.1 program (Statsoft, Cracow, Poland). The results were considered statistically significant at *p* < 0.05. Statistical analysis was mainly carried out using parametric methods, but if the data did not fulfill the assumptions of these methods, then they were further transformed. The data distribution shapes and deviations from the shape of the normal distribution were analyzed by the Shapiro–Wilk test. The significance in differences in mean values between the groups was analyzed by Student’s *t*-test for parametric data and Mann–Whitney *U* test for nonparametric data. Correlation analysis was performed using Spearman’s rank correlation test. The analyzes were conducted taking into account the cut-off points for BEI at levels 2 and 4. This means that the patients were divided into two groups for the purposes of statistical analysis taking into account the BEI cut-off points at the level of 2 (BEI < 2 and BEI ≥ 2) and into two groups taking into account the BEI cut-off points at the level of 4 (BEI ≤ 4 and BEI > 4).

## Results

A total of 105 patients (80 women and 25 men) were included in the study. All were Caucasians and aged 18–63 years (mean age ± standard deviation (SD): 33.43 ± 10.8 years). Of the 105 participants, 75 (71.43%) presented with SB (BEI ≥ 2), with 50 patients (47.62%) having severe SB (BEI > 4), while the remaining 30 (28.57%) did not (Table 1).

**Table 1:** Descriptive statistics of all studied parameters

Parameter	Mean	Median	Minimum	Maximum	SD	Shapiro–Wilk (normality)
BEI	4.44	3.90	0.20	16.20	3.451	*W* = 0.90329, *p* = 0.00000
BBI	5.13	3.30	0.00	23.80	4.974	*W* = 0.79329, *p* = 0.00000
Tonic bruxism	1.63	1.20	0.00	7.60	1.386	*W* = 0.83206, *p* = 0.00000
Mixed bruxism	0.94	0.80	0.00	5.00	0.831	*W* = 0.86313, *p* = 0.00000
Phasic bruxism	1.96	1.00	0.00	11.20	2.339	*W* = 0.86313, *p* = 0.00000
TPH1	116.69	78.21	5.30	631.63	120.488	*W* = 0.78568, *p* = 0.00000
DDC	1330.09	1116.96	329.69	6241.60	874.653	*W* = 0.80587, *p* = 0.00000

*SD*, standard deviation; *BEI*, bruxism episodes index; *BBI*, bruxism burst index; *TPH1*, tryptophan hydroxylase 1 [ng/mL]; *DDC*, aromatic l-amino acid decarboxylase [pg/mL]

### Enzyme levels and SB

**Fig. 1:**
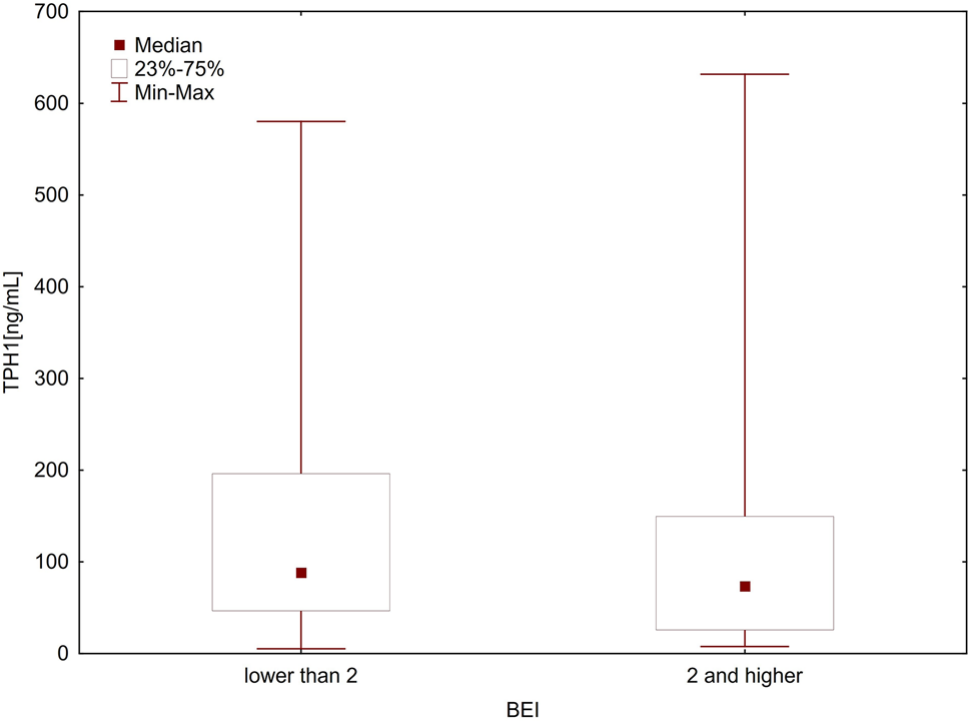
Comparison of TPH1 levels in groups with BEI ≥ 2 and BEI < 2. *TPH1*, tryptophan hydroxylase 1 [ng/mL]; *BEI*, bruxism episodes index

**Fig. 2:**
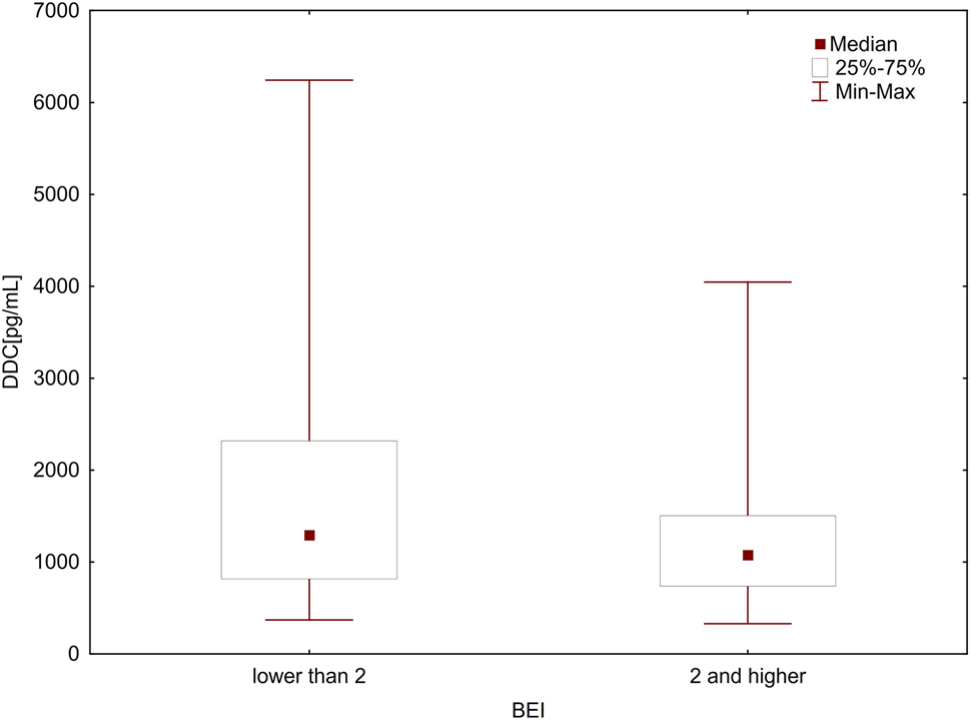
Comparison of DDC levels in groups with BEI ≥ 2 and BEI < 2. *DDC*, aromatic l-amino acid decarboxylase [pg/mL]; *BEI*, bruxism episodes index

**Fig. 3:**
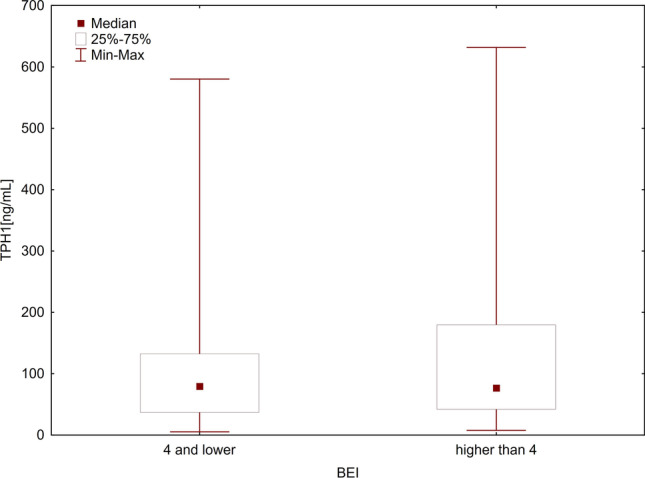
Comparison of TPH1 levels in groups with BEI ≤ 4 and BEI > 4. *TPH1*, tryptophan hydroxylase 1 [ng/mL]; *BEI*, bruxism episodes index

**Fig. 4:**
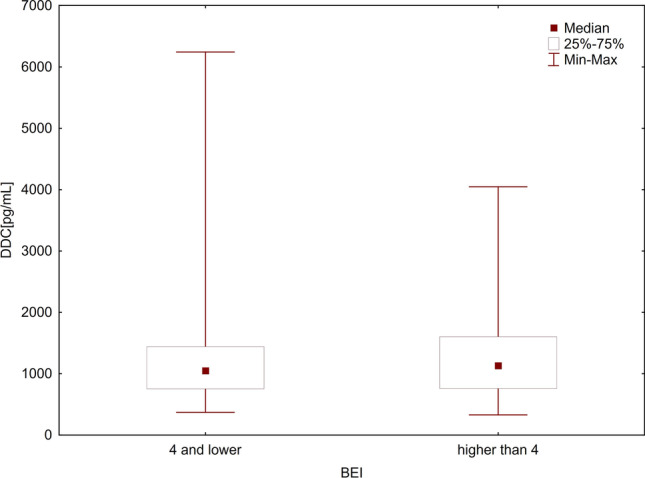
Comparison of DDC levels in groups with BEI ≤ 4 and BEI > 4. *DDC*, aromatic l-amino acid decarboxylase [pg/mL]; *BEI*, bruxism episodes index

The relationships between SB and the levels of TPH1 and DDC levels were analyzed in two ways. First, the enzyme levels were analyzed considering the two cutoff points of BEI (BEI = 2 and BEI = 4). For this purpose, the patients were divided into two groups: those without SB (BEI < 2) and those with SB (BEI ≥ 2). The differences in the levels of both TPH1 and DDC between the groups were not found to be significant using the Mann–Whitney *U* test (*p* = 0.379 and *p* = 0.249, respectively) (Table 2, Figs. 1 and 2). Then, the patients were again divided into two groups: those without SB or with mild-to-moderate SB (BEI ≤ 4) and those with severe SB (BEI > 4). Even in this case, the Mann–Whitney *U* test showed no statistically significant differences in the levels of both TPH1 and DDC between the groups (*p* = 0.746 and *p* = 0.498, respectively) (Table 2, Figs. 3 and 4).

**Table 2:** TPH1 and DDC levels in relation to BEI cutoff points

Enzyme	BEI	N	Mean	Median	Minimum	Maximum	SD	*U*	*Z*	*P*-value
TPH1	≥ 2	75	107.99	73.59	7.72	631.63	109.326	1000.50	–0.880	0.379
	< 2	30	138.45	88.26	5.30	580.20	144.492			
	≤ 4	55	111.75	79.55	5.302	580.20	118.977	1324.00	0.32	0.746
	> 4	50	122.12	76.65	7.725	631.63	123.105			
DDC	≥ 2	75	1227.68	1077.58	329.69	4046.61	690.517	962.00	–1.153	0.249
	< 2	30	1586.12	1292.88	371.50	6241.60	1196.609			
	≤ 4	55	1335.81	1049.01	371.503	6241.60	988.484	1269.00	0.68	0.498
	> 4	50	1323.79	1132.80	329.685	4046.61	739.478			

*TPH1*, tryptophan hydroxylase 1 [ng/mL]; *DDC*, aromatic l-amino acid decarboxylase [pg/mL]; *BEI*, bruxism episodes index; *SD*, standard deviation

**Fig. 5:**
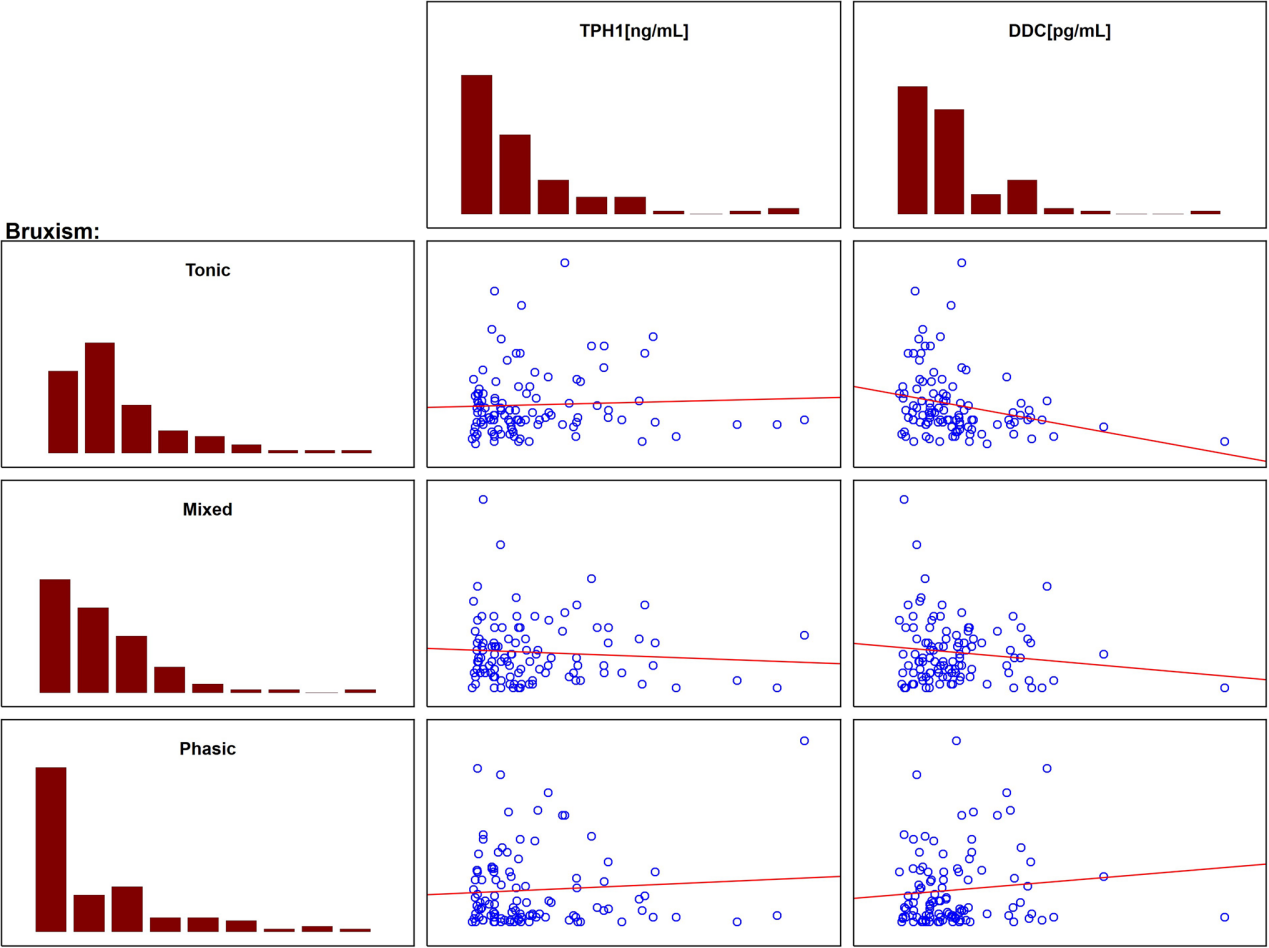
Correlations between TPH1 and DDC levels and SB parameters. *TPH1*, tryptophan hydroxylase 1 [ng/mL]; *DDC*, aromatic l-amino acid decarboxylase [pg/mL]

Next, the correlation between the levels of TPH1 and DDC and the SB parameters was studied. A statistically significant negative correlation between tonic SB episodes and DDC levels was revealed by Spearman’s rank correlation coefficient test (*p* = 0.0012). Other correlations were statistically insignificant (*p* > 0.05 for all comparisons). Table 3 and Fig. 5 present all the studied correlations.

**Table 3:** Correlations between TPH1 and DDC levels and SB parameters

Parameter	Enzyme	Spearman’s *r*-value	*P*-value
BEI	TPH1	–0.008	0.939
	DDC	–0.062	0.530
Tonic bruxism	TPH1	0.072	0.4635
	DDC	–0.313	0.0012
Mixed bruxism	TPH1	–0.072	0.465
	DDC	–0.040	0.687
Phasic bruxism	TPH1	–0.064	0.519
	DDC	0.089	0.369

*TPH1*, tryptophan hydroxylase 1 [ng/mL]; *DDC*, aromatic l-amino acid decarboxylase [pg/mL]; *SB*, sleep bruxism; *BEI*, bruxism episode index

## Discussion

Our previous genetic study showed that the serotonin neurotransmission pathway may be linked with predisposition to and pathogenesis of SB [11]. The results also indicated that severe bruxers most likely had lower blood levels of serotonin. However, the mechanism by which the serotonin neurotransmission pathway affects SB is not clearly understood yet.

In the human body, serotonin synthesized in the gastrointestinal tract regulates intestinal movements and vasoconstriction (when taken up by blood platelets), while that synthesized in the central nervous system controls feeding [21], sexual behaviors [22], thermoregulation [23], aggression [24], endocrine regulation [25], pain [26], memory and emotion [27], sleep [28], and motor activity [29]. Thus, it can be assumed that SB, which is defined as motor activity, could also be possibly regulated by serotonin [1–4]. Despite the fact that serotonin is synthesized from tryptophan, L-tryptophan supplementation was not reported to decrease the intensity of SB [16]. In the present study, we hypothesized that another stage of the serotonin synthesis pathway may be associated with the decreased level of serotonin observed in patients with severe SB. Perhaps, the levels of enzymes involved in serotonin synthesis may be responsible. Thus, this exploratory research was performed to evaluate the levels of TPH1 and DDC in individuals with SB diagnosed using vPSG [1–4] and compare them with that of individuals not presenting with SB.

The results of this study showed no statistically significant differences in the levels of TPH1 and DDC between bruxers and nonbruxers. No statistically significant correlations were found between the levels of TPH1 and bruxism parameters such as BEI as well as a phasic, tonic, and mixed bruxism. Similarly, there was no statistically significant correlation between the DDC levels and BEI as well as phasic and mixed bruxism. However, Spearman’s rank correlation coefficient test indicated that a statistically significant negative correlation existed between tonic bruxism and the levels of DDC.

To the best of our knowledge, this is the first study to evaluate if there is any relationship between the enzymes involved in the serotonin synthesis pathway and the pathogenesis of SB (potentially correlated with a decreased serotonin level). The results suggested that the levels of enzymes that play an important role in serotonin synthesis did not have any impact on the occurrence and severity of SB; however, the involvement of the serotonin pathway in SB pathogenesis seemed probable. Although the study did not show any statistically significant correlations, its findings may be an important contribution to further research on the relationship between the serotonin pathway and SB. They also indicate the need to analyze the reason for low serotonin levels in SB patients, which may be related not only to serotonin synthesis but also to processes associated with the serotonin neurotransmission pathway. Minakuchi et al. [30, 31] evaluated the correlation between the frequency of SB and serotonin transporter-driven serotonin uptake in platelets. Their results demonstrated a significantly higher serotonin uptake by serotonin transporters in peripheral platelets in the case of controls as compared to subjects with severe SB [30]. Similar findings were reported by Minakuchi et al. in another study [31], but unfortunately both their studies had serious limitations, including a very small number of participants and lack of the use of vPSG for the diagnostic purpose [30, 31]. Considering the results of the present study, this suggests that there is a need to thoroughly analyze serotonin-dependent neurotransmission in the context of SB. The negative correlation between tonic bruxism and DDC reported in this study should also be considered in the analysis. As previous studies reported that SB was correlated with sleep-related breathing disorders [5, 6] (especially obstructive sleep apnea) and tonic muscle contractions were possibly involved in SB as well as sleep-related breathing disorders [6], the relationship between increased contractions of tonic masticatory muscles and decreased levels of DDC should be further explored.

The present study was conducted on a large population and used vPSG which is the most appropriate objective method for diagnosing SB. However, it has some potential limitations. First, only a single-night vPSG examination was conducted and there were no patients without clinical suspicion of SB or sleep disorders included in this study due to the restrictions of the Polish healthcare system. Second, the study is exploratory in nature, and hence, the topic needs further research.

## Conclusion

The study showed that the levels of enzymes that are crucial for serotonin synthesis (TPH1 and DDC) did not seem to influence SB. However, it indicated the need for further research, taking into account not only the process of serotonin synthesis but also the effect of serotonin-dependent neurotransmission on SB.
